# Opening clinical trial data: are the voluntary data-sharing portals enough?

**DOI:** 10.1186/s12916-015-0525-y

**Published:** 2015-11-11

**Authors:** Nophar Geifman, Jennifer Bollyky, Sanchita Bhattacharya, Atul J. Butte

**Affiliations:** Institute for Computational Health Sciences, University of California San Francisco, Mission Hall, 550 16th Street, 4th Floor, San Francisco, CA 94158-2549 USA; Sean N. Parker Center for Allergy Research, Department of Pediatrics, Division of Allergy, Immunology & Rheumatology, Stanford University School of Medicine, Stanford, CA USA

**Keywords:** Clinical data, Clinical trials, Meta-analysis, Open access, Reanalysis

## Abstract

Data generated by the numerous clinical trials conducted annually worldwide have the potential to be extremely beneficial to the scientific and patient communities. This potential is well recognized and efforts are being made to encourage the release of raw patient-level data from these trials to the public. The issue of sharing clinical trial data has recently gained attention, with many agreeing that this type of data should be made available for research in a timely manner. The availability of clinical trial data is most important for study reproducibility, meta-analyses, and improvement of study design. There is much discussion in the community over key data sharing issues, including the risks this practice holds. However, one aspect that remains to be adequately addressed is that of the accessibility, quality, and usability of the data being shared. Herein, experiences with the two current major platforms used to store and disseminate clinical trial data are described, discussing the issues encountered and suggesting possible solutions.

## Background

Approximately 30,000 clinical trials are conducted annually worldwide, producing a large volume of raw patient-level data. Sharing of such data would allow the scientific community to independently verify published results and evaluate new hypotheses, either by extending the analysis of data from a clinical trial or by combining data from different trials. The unavailability of original research data is a known and significant barrier to reproducibility. Further, the enormous quantity of raw, individual-level clinical and high-throughput mechanistic assay data available should function as a springboard for scientific advances and development of new techniques in clinical informatics. With this in mind, recent market and regulatory forces have been driving initiatives to release such data to the public. Nevertheless, for this potential to be realized, the data have to be accessible and usable.

Many government agencies, research groups, and pharmaceutical companies have begun implementing mechanisms for data sharing [1]. While there is ongoing discussion in the community over the key issues and risks of sharing data [2], most sharing of clinical trial data by pharmaceutical companies is currently performed on a voluntary basis through web-based portals where access is carefully controlled. Whether this voluntary, controlled sharing will be sufficient to meet the goals of transparency and reanalysis is yet to be assessed. One aspect that has not been thoroughly addressed is that of the accessibility, quality, and usability of the data being shared. From our experiences in seeking access to patient-level clinical trial data through the two current major platforms used to store and disseminate these data, the process is still very cumbersome and lengthy and, when data are provided, they are not readily useable.

## Our experience in the pursuit of open clinical trial data

One of the largest of such platforms is ClinicalStudyDataRequest.com, which, to date, allows users to browse over 2,100 clinical trials sponsored by 12 different pharmaceutical entities, select those of interest and, following the platform’s protocol, request access to the data [3]. However, the process of gaining access to the data is time consuming and demotivating. A detailed research proposal providing a lay summary, study design, primary and secondary endpoints, statistical analysis plan, and publication plan needs to be provided. The submitted proposal is reviewed by an independent research panel which decides whether to approve the request for data. This requirement for review prior to allowing data access is considered necessary to ensure that data are released only to responsible and qualified parties, thus avoiding misinterpretation of the data and publication of misleading, erroneous results. Once approved, a data-sharing agreement is sent to all trial sponsors and returned to the requesting researcher for signature. Following sign-off, trial sponsors prepare, anonymize, and make the data available via an analysis platform. Thus, the original data are never actually shared with the researcher and analysis is limited to that which can be conducted via the platform.

**Fig. 1:**
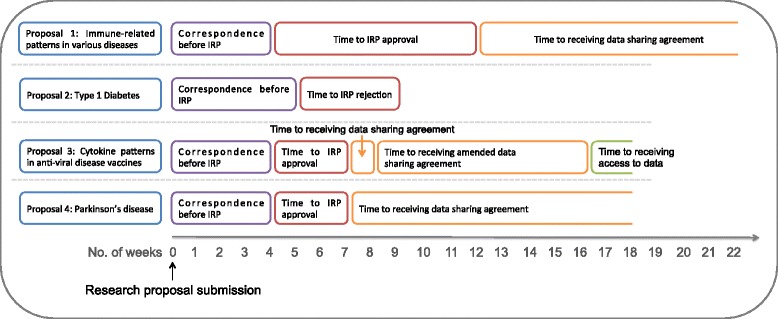
Timelines for data request research proposals submitted to ClinicalStudyDataRequest.com. Four research proposals were submitted to the site requesting access to data in different medical areas. Each box represents a different stage in the proposal evaluation and granting of access to data. IRP, Independent research panel

Four proposals, each requesting data for different research questions and from different trials, two of which combined data from several sponsors, were submitted to ClinicalStudyDataRequest.com (from December 2014 to January 2015). Each submission was followed by numerous exchanges of correspondence asking for additional information not initially requested, such as the resume of the team’s statistician or confirmation of intent to conduct the analysis within the data access system, resulting in a processing time of over 2 months from submission to approval or rejection. At the time of writing, over 5 months after initial submission, two requests were still pending their data-sharing agreement, one had been denied and was pending re-submission, and the fourth was still waiting to receive access to data (the timeframes are illustrated in Fig. 1). While every step in this process could be argued to be necessary to prevent misuse and protect patient privacy, our experience from four attempts to obtain data through the site has led us to conclude that the process is less than ideal and would benefit from improved efficiencies to better foster advances in research, while minimizing the inherent risks of data sharing.

Faster data acquisition, within days, was achieved via the Project Data Sphere database (projectdatasphere.org) which stores, shares, and allows analysis of patient level phase III cancer trial data [4]. The database currently holds information from over 50 cancer clinical trials and, after registration and submission of a simple research proposal, which was swiftly approved, all data on the site were made available for either download or analysis on an online SAS platform. Nevertheless, whilst obtaining the data was fast and relatively straightforward, other than when using the restrictive online tools, the data were not readily usable. For the studies available on the site, data schemas were not uniform, making integration and comparison of data from different studies, such as meta-analyses, difficult. Further, table and variable names were uninformative. Long annotation files had to be manually read to assess what data were available and, for at least five studies, no annotation files were provided. Of even greater concern was the fact that only data pertaining to the control, placebo, or comparator arms were made available, making the possibility of reanalysis of studies, such as those which failed, impossible.

While this is merely the dawn of a new age of open clinical trial data and, understandably, hurdles toward implementation of safe and efficient clinical trial data-sharing platforms are expected, examples of effective platforms already exist. One initiative, led by the National Institute of Allergy and Infectious Diseases, is the Immunology Database and Analysis Portal (ImmPort; www.immport.org) data-sharing platform [5]. In the short time since its implementation, over 140 datasets (including 36 clinical trials, as of October 2015) from various research consortia were made publicly available in an easily accessible and readily useable way. Access to the data stored in ImmPort, most of which is parsed, annotated and structured, is made available immediately after registration to the site. With hundreds of downloads per month, ImmPort is an important source of raw data and protocols from clinical trials, mechanistic studies, and novel methods for cellular and molecular measurements.

Our experiences, albeit based on a small sample, have led us to the conclusion that, although the pharmaceutical industry is making some effort, it is not yet highly motivated to share clinical trial data in a timely manner. Clinical trial data are complex and comprise multiple aspects; a widespread adoption of standards for publishing and sharing such data would prevent many of the issues we encountered. The recently published Institute of Medicine’s report [1] provides guidelines for clinical trial data sharing. Adoption of these guidelines, along with standards for data representation, such as standard data table templates and standard vocabularies, would greatly assist in making open clinical trial data useful to the community. An agreed-upon protocol for the approval of data requests and reasonable time frames for the process would also shorten the path from resource to research. Naturally, newer and acceptable standards should be developed in collaboration with the industry providers. We recommend that action be taken to establish community-wide standards and guidelines and a platform for communication between researchers and providers regarding data safety, access, and usability concerns. Additionally, a scheme to increase the industry’s motivation for data sharing, rewarding companies that make data readily accessible (e.g. by extension of the duration of patent exclusivity for the tested drug [6] or by participation in publications related to re-use of the data) and covering the costs of this process, should also be considered.

## Conclusions

Better sharing of clinical trial data holds great potential for the scientific and patient communities. Collaborative establishment of data standards and processes for data sharing and acquisition would greatly accelerate the progress of research based on this rich data source.
